# Acquisition of Torrefied Biomass from Jerusalem Artichoke Grown in a Closed Circular System Using Biogas Plant Waste

**DOI:** 10.3390/molecules25173862

**Published:** 2020-08-25

**Authors:** Szymon Szufa, Piotr Piersa, Łukasz Adrian, Jan Sielski, Mieczyslaw Grzesik, Zdzisława Romanowska-Duda, Krzysztof Piotrowski, Wiktoria Lewandowska

**Affiliations:** 1 Faculty of Process and Environmental Engineering, Lodz University of Technology, Wolczanska 213, 90-924 Lodz, Poland; piotr.piersa@p.lodz.pl (P.P.); lukasz.adrian@p.lodz.pl (Ł.A.); 2 Department of Molecular Engineering, Lodz University of Technology, Wolczanska 213, 90-924 Lodz, Poland; jan.sielski@p.lodz.pl; 3 Department of Variety Studies, Nursery and Gene Resources, Research Institute of Horticulture, Str. Konstytucji 3 Maja 1/3, 96-100 Skierniewice, Poland; mieczyslaw.grzesik@inhort.pl; 4 Department of Plant Ecophysiology, Faculty of Biology and Environmental Protection, University of Lodz, Str. Banacha 12/16, 92-237 Lodz, Poland; zdzislawa.romanowska@biol.uni.lodz.pl (Z.R.-D.); k_piotrow@o2.pl (K.P.); 5 Faculty of Chemistry, University of Lodz, Tamka 12, 91-403 Lodz, Poland; wiktoria.lewandowska.uni.lodz@gmail.com

**Keywords:** torrefaction, Jerusalem artichoke, biofuel, energy crops, agiculture

## Abstract

The aim of the research was to investigate the effect of biogas plant waste on the physiological activity, growth, and yield of Jerusalem artichoke and the energetic usefulness of the biomass obtained in this way after the torrefaction process. The use of waste from corn grain biodigestion to methane as a biofertilizer, used alone or supplemented with Apol-humus and Stymjod, caused increased the physiological activity, growth, and yield of Jerusalem artichoke plants and can limit the application of chemical fertilizers, whose production and use in agriculture is harmful for the environment. The experiment, using different equipment, exhibited the high potential of Jerusalem artichoke fertilized by the methods elaborated as a carbonized solid biofuel after the torrefaction process. The use of a special design of the batch reactor using nitrogen, Thermogravimetric analysis, Differential thermal analysis, and Fourier-transform infrared spectroscopy and combustion of Jerusalem artichoke using TG-MS showed a thermo-chemical conversion mass loss on a level of 30% with energy loss (torgas) on a level of 10%. Compared to research results on other energy crops and straw biomass, the isothermal temperature of 245 °C during torrefaction for the carbonized solid biofuel of Jerusalem artichoke biomass fertilized with biogas plant waste is relativlely low. An SEM-EDS analysis of ash from carbonized Jerusalem artichoke after torrefaction was performed after its combustion.

## 1. Introduction

One of the greatest global problems is increasing energy consumption, which, in the face of the need to limit the use of fossil fuels, forces the development of crops that will produce the maximum yield of biomass, which could be converted into energy fuel using modern technologies [1]. For this reason, research is necessary to select plants having a high potential of biomass and energy yield on poor soils and to develop plant cultivation technologies that, in addition to high biomass yield and energy efficiency, will be conducive to the environment by reducing the use of chemistry in agricultural production and will strengthen energy security [2].

Compared to other renewable energy sources, biomass provides continuous electricity generation, and is the only widespread source of renewable heat. Biomass co-firing and biomass combustion will contribute to the reduction of CO_2_ and SO_2_ emissions, support sustainable development, and increase energy security and regeneration of rural areas, due to the increase of forestry and agricultural activity and the provision of heat and electrical energy production. To increases the biomass share up to a 30% or even 40% caloric value, the biomass particles must be milled down to sizes where high caloric values can be expected. There are many different biomass pre-treatment methods that can be used to convert it into more coal-like matter. There are a number of barriers to overcome in order to expand the exploitation of biomass for heat and eleclricity production. One of them concerns the limitations connected to biomass fuel characteristics [3,4,5].

When coal is compared with wood biomass, which are both still the dominant solid fuels in heat and electricity production in Poland, the inferior properties of biomass are often revealed. Wood biomass fuel has in most cases a high moisture content, resulting in storage complications, such as self-heating and biological degradation, and lower energy densities. It is also a bulkier fuel (with poorer transportation and handling characteristics), and it is more tenacious (the fibrous nature of biomass makes it difficult to reduce it to small homogeneous particles). The biomass properties mentioned above have negative impacts during energy thermal conversion, such as gasification and lower combustion and co-firing efficiencies [6].

Among the methodologies that can be applied to improve the properties of plant biomass and make it a more coal-like material, torrefaction (biomass carbonization) seems to provide many advantages. Carbonization, or torrefaction, is a thermal degradation of biomass structures, which occurs by heating them without air contact under atmospheric pressure. It removes low-weight organic volatile components and moisture as well as depolymerizes the long polysaccharide chains of biomass. This kind of process of wood carbonization is quite a complex research subject due to the fact that wood contains different fractions. Wood cells are built from microfibrils, bundles of cellulose molecules ‘coated’ with hemicellulose. Another component of wood biomass is lignin, which is deposited between microfibrils and in some types of biomass in the amorphous regions of the microfibril. All of those three fractions exhibit different thermal behavior [7]. The product of torrefaction is a hydrophobic solid fuel with greatly increased grindability and energy density (on a mass basis). More importantly, the energy requirement for processing the torrefied biomass decreases and it no longer requires additional separate handling facilities when we co-combust new fuel with coal in operating power plants. It is suggested that torrefied biomass can be compacted into high-grade pellets with substantially superior fuel properties compared with standard wood pellets from un-treated biomass. The carbonization process can be combined together with the drying and pelletization process, with both energy end economical benefits. The biomass torrefaction process has proved suitable for feedstock for flow gasification, which has not been considered feasible before for raw biomass. This is due to the fact that carbonized biomass forms more solid fuel spherical-shaped particles during milling or grinding than raw biomass. To produce high caloric value carbonized solid biofuel, which will have a reasonable price (lower torrefaction process costs can be achieved by using superheated steam), with better physical-chemical properties before thermo-chemical conversion, such as a hydrophobic nature, low moisture content, and better grindability, several process conditions have to be optimal. These include a 30% loss of the mass and 10% loss in energy in volatile matter plus a low as possible temperature and residential time in the reactor, which can ensure successful sale on the Polish and European market.

Important parameters in the choice of highly efficient plant species for energy and torrefaction purposes comprise their physiological properties, decisive for high biomass yield and the amount of energy obtained. Jerusalem artichoke (*Helianthus tuberosus* L.) meets these requirements and it is well adapted to the conditions of central Europe. The biomass of this species is an important raw material for the production of bioethanol, for burning to obtain heat energy, and is widely used in the feed, food, and medical industries [8,9,10].

A serious problem in the production of energy plants is the excessive use of artificial fertilizers and pesticides, which pollute the environment. There is a resulting need to decrease their volume without reducing the yield potential. Replacement of chemical fertilization by natural fertilizers, including waste from biogas plants, seems to be one of the ways to address this problem. Due to the fact that this waste contains the majority of nutrients necessary for plant growth, it seems that their use in agriculture can support soil fertilization. At the same time, this solves the problem of utilization and storage, which is dangerous for the environment. The problem is, however, that the fertilizing value of this waste and its impact on plant physiological activity depends on the type of biodegradable biomass, which requires separate research into the methods of its application and management. These difficulties become serious because biogas plants turn out to be a fast developing branch of energy production and they use of different raw materials. Alburquerque et al. [11], Jasiulewicz and Janiszewska [12], and Łagocka et al. [13] indicated that, given the environmental risks and benefits, the use of this waste in agriculture is most rational, provided that methods for its use are developed. The limitation of environmentally harmful synthetic fertilization by the use of biogas plant waste seems to be very important, as has been similarly demonstrated in the case of microalgae and water plants from the family *Lemnaceae*, which applied to medium enabled a reduction of the recommended artificial fertilizer doses [14,15,16,17]. The use of waste from biogas plants for fertilizing purposes is part of the strategy of the circulating production of energy plants in which waste becomes a raw material in the next crop cycle. Another unknown problem is the usefulness of the biomass energy produced from the waste of a biogas plant and the possibility of its torrefaction for energy purposes. The world literature available on these issues, especially concerning agricultural management of waste from the corn grain biodigestion to the methane process together with preparations stimulating growth, their influence on physiological processes that regulate plant energy properties, and the development of torrefaction technologies converting such produced biomass into energy fuel, is hard to find. In the majority of cases, it refers to the waste produced by specific biogas plants and to the raw materials used there [18]. Additionally, the possibilities of torrefaction of the biomass produced on this waste, as well as its energy properties so far are not known.

The purpose of this work was to describe the impact of waste from the corn grain biodigestion to methane process, used as biofertiliser either separately or together with Apol-humus and Stymjod, on the growth, yield, and physiological properties of Jerusalem artichoke biomass and the possibility of converting it into valuable energy fuel using the torrefaction processes.

## 2. Results

**Figure 1:**
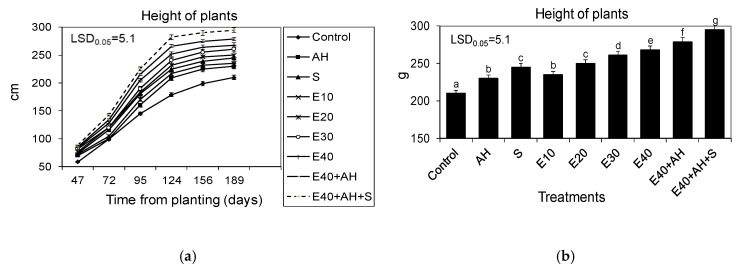
Kinetics of the growth (**a**) and final height of Jerusalem artichoke plants (**b**) cultivated in a field and fertilized with liquid, non-centrifuged waste from corn grain digestion to methane (E10, 40 m^3^ ha^−1^), Apol-humus (AH; 10 L ha^−1^), and Stymjod (S; 5 L ha^−1^). The data marked with the same letters are not significantly different, according to the Newman–Keuls multiple range test at an alpha level of 0.05. The data presented are the average over the years and 10 plants in each repetition of a particular experimental variant.

**Figure 2:**
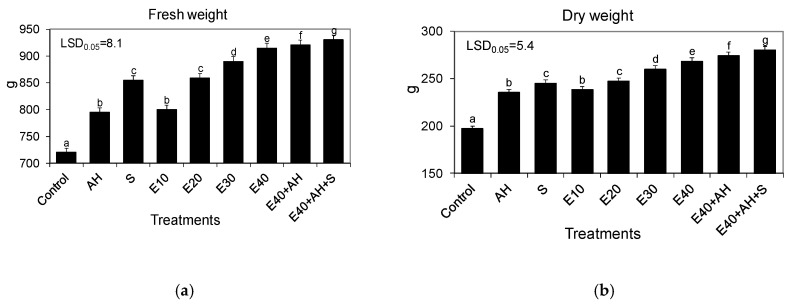
Fresh (**a**) and dry biomass (**b**) of one Jerusalem artichoke plant cultivated in a field and fertilized with liquid non-centrifuged waste from corn grain digestion to methane (E10, 40 m^3^ ha^−1^), Apol-humus (AH; 10 L ha^−1^), and Stymjod (S; 5 L ha^−1^). The data marked with the same letters are not significantly different, according to the Newman–Keuls multiple range test at an alpha level of 0.05. The data presented are the average over the years and five plants in each repetition of a particular experimental variant.

The waste from the corn grain biodigestion to methane process had a beneficial influence on the growth biomass yield and physiological activity of Jerusalem artichoke. All quantities of applied waste accelerated the Jerusalem artichoke growth and biomass yield, with 30–40 m^3^ ha^−1^ being the most favorable for plant development. The positive impact of this natural fertilizer on growth was enhanced by the additional application of Apol-humus to soil (10 L ha^−1^) and, furthermore, to a higher degree by supplementary double plant spraying with Stymjod (5 L ha^−1^) (Figure 1 and Figure 2).

Biogas plant waste, applied alone or supplemented with Apol-hymus and Stymjod, increased the activity of acid and alkaline phosphorylases, RNase, and dehydrogenases. The activities of these enzymes were closely related to the increasing doses of fertilizers (Table 1).

**Figure 3:**
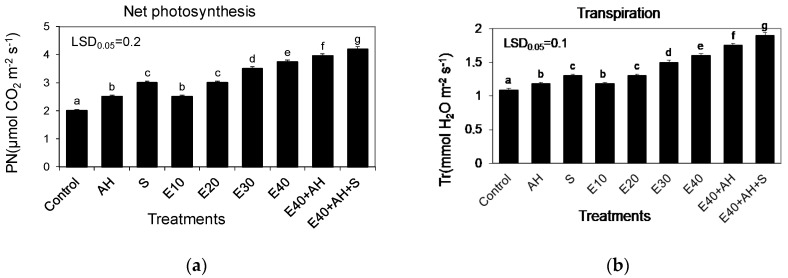

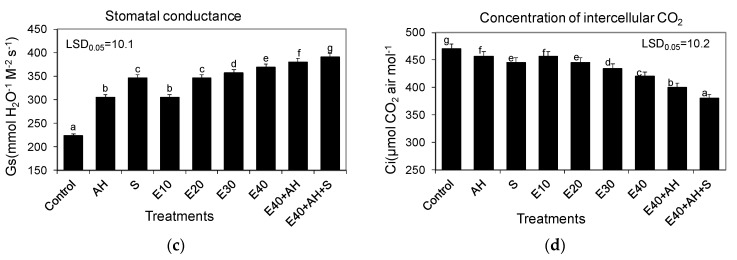
Gas exchange in the leaves of Jerusalem artichoke plants (Net photosynthesis (**a**), Transpiration (**b**), Stomatal conductance (**c**), Concentration of intercellular CO_2_ (**d**)) cultivated in a field and fertilized with liquid non-centrifuged waste from corn grain digestion to methane (E10, 40 m^3^ ha^−1^), Apol-humus (AH; 10 L ha^−1^), and Stymjod (S; 5 L ha^−1^). The data marked with the same letters are not significantly different, according to a Newman–Keuls multiple range test at an alpha level of 0.05. The data presented are the average over the years and 10 plants in each repetition of a particular experimental variant.

**Figure 4:**
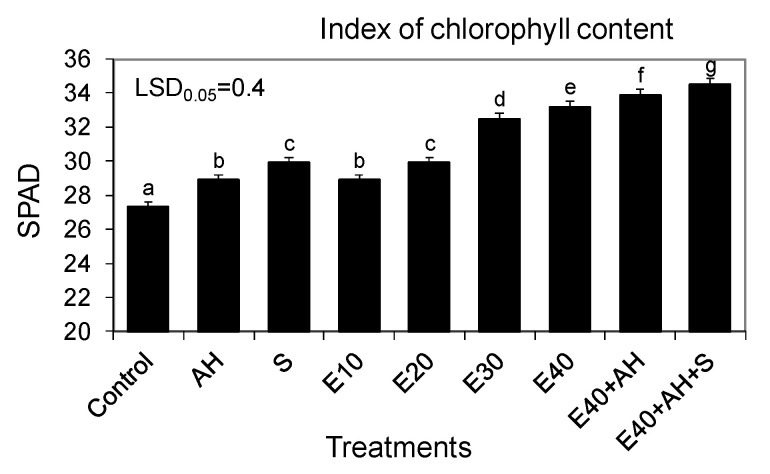
Index of the chlorophyll content in leaves of the Jerusalem artichoke plants cultivated in a field and fertilized with liquid non-centrifuged waste from corn grain digestion to methane (E10, 40 m^3^ ha^−1^), Apol-humus (AH; 10 L ha^−1^), and Stymjod (S; 5 L ha^−1^). The data marked with the same letters are not significantly different, according to a Newman–Keuls multiple range test at an alpha level of 0.05. The data presented are the average over the years and 10 plants in each repetition of a particular experimental variant.

The established correlations between the favorable changes in plant growth and biomass yield and the fertilizer doses studied were also confirmed by the proportionally increased activity of gas exchange and the index of the chlorophyll content in leaves. The growing doses of waste, used alone or supplemented with Apol-humus and Stymjod, also increased the index of the chlorophyll content, net photosynthesis, transpiration, and stomatal conductance, and decreased intercellular CO_2_ concentration, inversely proportional to the above-mentioned three parameters of gas exchange. These relationships between the doses used of waste and parameters of gas exchange and the index of the chlorophyll content were similar to those observed between the amount of the fertilizers used and the plant growth and biomass yield (Figure 1, Figure 2, Figure 3 and Figure 4).

The use of biogas plant waste slightly increased the heat of combustion in the analytical state and calorific value in the working state and decreased the ash content in the plants. Table 2 presents the results of the main experiment using a batch reactor for conducting a Jerusalem artichoke torrefaction process in a nitrogen atmosphere. The most important observation is that under 245 °C and a residential time of 13 min, the mass reduction was the closest one of all experimental results to 30%, which is due to the large amount of literature on the most optimal ratio.

In Table 3, the results of a proximate analysis of Jerusalem artichoke before and after the thermo-chemical conversion process are presented. It is quite clear that the C% (weight) in the biomass thermo-chemical process of bio-products increases in tandem with an enhancement in the Jerusalem artichoke torrefaction process temperature. This was contrary to the weight percentages of C_a_H and O, which showed a decreasing trend. From the above mechanism, it is clear that dehydration takes place as well as de-carbonization during the Jerusalem artichoke torrefaction process. This clearly shows that the emission of CO_2_, CO, or H_2_O will result in a decrease in the H and O contents of torrefied biomass. The rising % of the C content was only due to a decrease in the O content.

**Figure 5:**
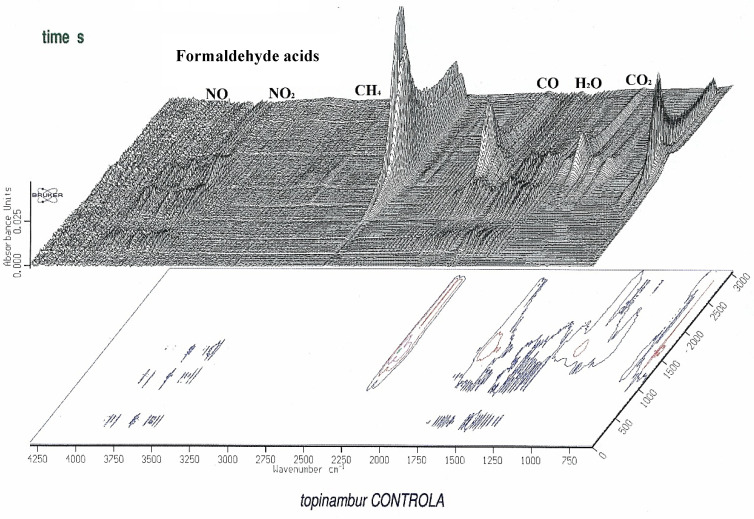
FTiR analysis of Jerusalem artichoke torrefaction by-products: torgas during the torrefaction process under 245 °C.

**Figure 6:**
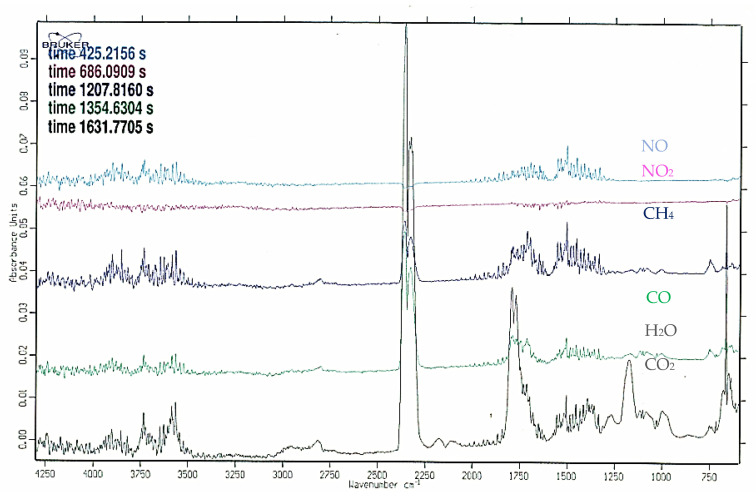
FTiR analysis of Jerusalem artichoke torrefaction by-products: torgas during the torrefaction process under 245 °C.

An FTiR analysis during the thermogravimetric analysis of the Jerusalem artichoke torrefaction process under 245 °C for the production of carbonized solid biofuel shows what kind of volatile matter components are produced, so-called torgas: H_2_O, CO, CO_2_, CH_4_, and C_2_ (Figure 5 and Figure 6). 

**Figure 7:**
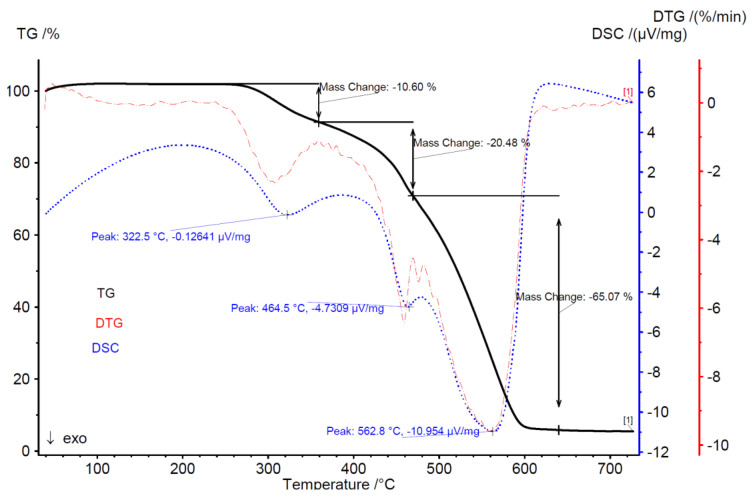
Thermogravimetric analysis of torrefied Jerusalem artichoke combustion process.

**Figure 8:**
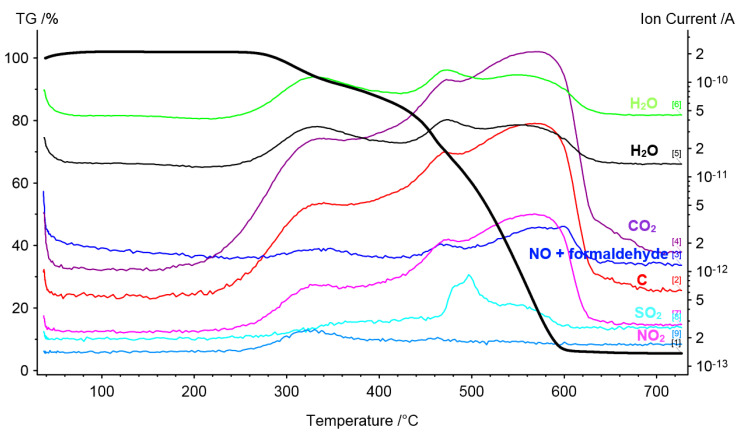
TGA-MS analysis of the Jerusalem artichoke combustion process.

**Figure 9:**
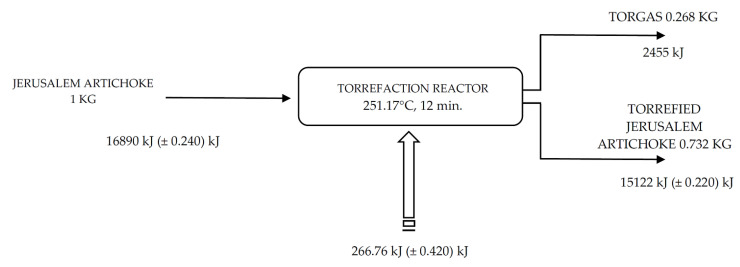
Mass and energy balance of the Jerusalem artichoke torrefaction process (*t* = 251.17 °C, 12 min).

**Figure 10:**
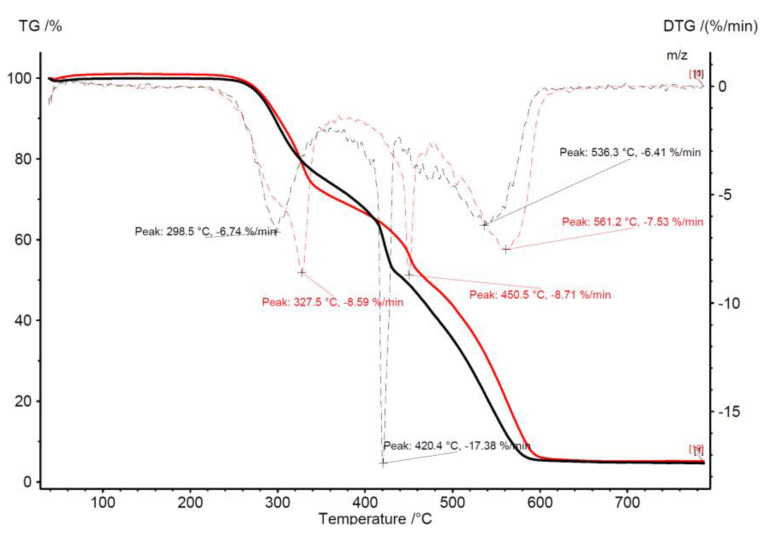
TGA-MS analysis of the torrefied Jerusalem artichoke combustion process.

Figure 7, Figure 8, Figure 9 and Figure 10 presents the thermogravimetric analysis of the combustion process of torrefied Jerusalem artichoke and TG-MS analysis, which shows what kind of component occurs during combustion. The colored lines represent what kind of volatile components are formulated during the combustion process: H_2_O, CO_2_, NO, SO_2_, NO_2_, formaldehyde, and C.

An elemental analysis shows that during the TG-MS analysis of the torrefaction process of Jerusalem artichoke under 245 °C in torgas, there is 0.1% of CH_4_, 0.05% of C_2_, 81.2% of CO_2_, and 18.6% of CO (Figure 8). Figure 9 represents the mass and energy balance of the Jerusalem artichoke torrefaction process using a batch reactor and in an inert atmosphere of nitrogen. It was calculated that 266.76 (±0.420) kJ is the external energy, which is neccessery to produce carbonized solid biofuel (with a 30% mass loss and 10% energy loss) from Jerusalem artichoke. The novelty of this research is based on the fact that there is a lack of publications describing the use of Jerusalem artichoke growing in Poland on low-class soils with the addition of wastes and biofertilizers as a feedstock for the torrefaction process with a focus on the torrefaction process’s optimal parameters for biofuel production and physical and chemical analysis of torrefied biofuels and uses ashes as carriers of C for biofertilisers (Table 4).

**Figure 11:**
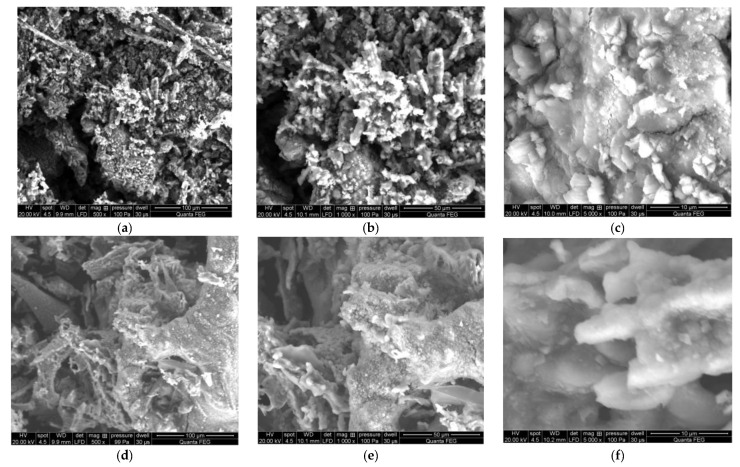
SEM-EDS microscopic images (**a**) 100 µm, (**b**) 50 µm, (**c**) 10 µm of ashes from torrefied Jerusalem artichoke after combustion and (**d**) 100 µm, (**e**) 50 µm, (**f**) 10 µm of ashes from untreated Jerusalem artichoke after combustion.

Torrefaction is a thermal pre-treatment step for biomass co-firing or biofuel combustion purposes, which takes place in a relatively low temperature range of 225–350 °C to produce a fuel with a bigger energy density mainly by the decomposition of hemicellulose fractions (Figure 11).

## 3. Discussion

Jerusalem artichoke is a very realistic high production potential energy plant that can be used to produce large amounts of biomass (aerial part) and biofuels (using tubers). The amount of yield depends primarily on the plant genotype and soil fertility. Biomass yield can even reach 110 t ha^−1^, including green mass of 75.6 t ha^−1^, and tubers of 32.4 t ha^−1^. The raw material for energy purposes can be tubers that can be used for the production of ethanol or biogas and above-ground parts used for biogas fabrication, burning, or for fuel briquettes and pellets. This plant grows well and produces large amounts of biomass in a wide range of conditions, including moderately compact, well-ventilated, nutrient-rich, and sufficiently moist soils. It can also be cultivated in worse positions for energy needs, especially if it is fertilized with biogas plant waste, as the research presented demonstrates [9].

The main goal of the research was to examine mechanisms controlling the torrefaction process, to determine selected torrefaction parameters of Jerusalem artichoke growing in low-class soils, and to determine the impact of the torrefaction process on the Jerusalem artichoke co-firing with coal process, including the level of pollution reduction. The main thesis of the research is the assumption that the torrefaction process causes an increase in the calorific value of carbonized Jerusalem artichoke, positively affects the improvement fuel values during carbonization, and favorably affects the process of co-firing torrefied biomass with coal, thus limiting a number of operational problems arising as a result of co-firing biomass with hard coal. This research is becoming important as it is commonly estimated that in 2050, about 75% of energy will come from renewable energy sources [19]. Therefore, it is necessary to undertake intensive actions to develop technology for sustainable energy crop production, its conversion into useful biofuel, and to limit the use of synthetic fertilizers, as their production and use in agriculture are energy intensive and cause pollution of the environment. The research performed showed that fertilization with the waste from corn grain biodigestion to methane in a dose of 10–40 m^3^ ha^−1^ of Apol-humus and Stymjod increased plant growth, biomass yield, gas exchange, enzymatic activity, and energy value of Jerusalem artichoke biomass proportionally to the fertilizer doses, as was shown in several labour-intensive tests. The correlations found were observed in all tests performed on physiological activity and they were similar regardless of the fact that these plants were cultivated for three subsequent years in podzolic soil. This indicates the proper selection of the plant assessment used tests to show the response of plants to the applied treatments and the possibility of obtaining positive effects from the developed methods of fertilization with waste irrespective of the climatic conditions. The tests implemented also explain the correlation between particular physiological events and their role in regulating growth and yield [20,21]. The changes observed in the kinetics of Jerusalem artichoke growth, biomass yield, gas exchange, and enzyme activity in relation to the fertilization methods demonstrated the positive impact of all used non-centrifuged waste doses, with 30–40 m^3^ ha^−1^ being the most favorable for plant growth and physiological activity [19].

The results obtained show that the amounts of nutrients contained in the waste used could influence the photochemical processes of photosynthesis, as was also demonstrated by Kalaji et al. [22] in corn and tomato. They showed that photosynthetic system activity, measured by chlorophyll fluorescence, is related to the nutrient content in plants. The research presented showed also that the increasing doses of waste applied to soil were proportionally related to a higher chlorophyll content in leaves and accelerated growth of plants. Chlorophyll content is the most widely used proxy for N content, as was demonstrated by the studies of Herrmann et al. [23], Homolová et al. [24], and Camino et al. [25]. According to Hamann et al. [26], measurement of the chlorophyll content and nitrogen balance can be a useful non-destructive method to estimate the physiological status of plants, as was found in young apple trees cultivated under water stress conditions.

Research shows that the applied waste from biogas plants influenced the activity of enzymes, which have a key impact on plant growth, biomass yield, physiological processes, and energy properties. Similarly, as in the case of gas exchange, it enhanced the activities of acid and alkaline phosphorylases, RNase, and dehydrogenase in a dose-dependent manner. The alkaline and acid phosphorylases are responsible for the distribution of phosphorus in plants and they catalyze the hydrolysis of organic phosphorus. They also regulate the mineralization potential of organic phosphorus, which can influence the biomass energy value [27]. Stimulation of RNase activity by the waste studied may play an important role in strengthening defense mechanisms in plant tissues, as was also observed in willow and corn plants under the influence of microalgae used as fertilizer. Dehydrogenases play a crucial role in respiration processes important for growth and biomass yield [21]. A close relationship between different fertilization methods, the enzyme activities studied, and plant development was also found in other energy plants, such as Virginia fanpetals, corn and willow treated with algae [17,21,28,29], and in some energy plants fertilized with sewage sludge and ash [30,31].

The studies indicated that enhanced fertility, resulting in higher gas exchange and enzyme activity, also slightly increased the heat of combustion in the analytical state and calorific value in the working state, as well as decreased the ash content in plants proportionally to the applied fertilizer doses. These properties are important when plants are produced for energetic purposes and show that Jerusalem artichoke fertilized with the waste from a biogas plant is suitable for this use. In line with the research of Kordas et al. [32], the results obtained state that increasing the dose of mineral fertilization contributed to the increase in the heat value of plant combustion.

The additional stimulating impact on plant development of the biopreparations added to the waste from the biogas plant in the combined treatments could be caused by humid acids and chitosan polymers contained in Apol-humus and by-nutrients, and humid acids and iodine present in Stymjod. The positive impact of humid acids on plant development has been described in the literature [33]. The favorable impact of chitosan on the development and health of plants under hydrothermal stress was revealed by Górnik et al. [34] in grapevines. The demonstrated stimulatory influence of Stymjod, used with the waste studied, on Jerusalem artichoke plant development could be an effect of macro- and microelements, humid acids, and above all of the presence of iodine [35,36]. Jeznach [36] demonstrated the positive influence of iodine on the cyto-morphological changes in cabbage and tomato, the enlarged diameter of phloem and xylem, and more frequent stomata opening, which resulted in increased gas exchange in the leaves. The application of iodine to cabbage enhanced its resistance to stress and the quantity of several elements in leaves [36]. According to Smoleń et al. [37], iodine application increased the content of phosphorus, potassium, and calcium and decreased the accumulation of iron in stored carrot roots. The intensified physiological processes found in vegetable crops under the influence of iodine could also occur in Jerusalem artichoke and influence its biomass energy properties and sensitivity to growing conditions.

The research presented indicates that biogas plant waste use in Jerusalem artichoke crops under different climate conditions subsequently not only allows a reduction in the doses of synthetic fertilizers that contaminate the surroundings but can also solve the serious problem of utilization and storage, which is expensive and dangerous for the environment [38,39]. Additionally, the use of this waste as a plant fertilizer is safer than sewage sludge, which may often contain harmful compounds that must be removed prior to its use in agriculture [40]. Research indicates that fertilization with the waste from a biogas plant enables a high yield of energy and biomass to be obtained, which could be used in its torrefaction, and may lead to a decrease in the recommended doses of artificial fertilizers, thus limiting environmental pollution. The highest fertilization level of Jerusalem artichoke enables a high quantity of fuels to be obtained as was found in sorghum, which can be a raw material for producing 8455 Nm^3^ of biogas ha^−1^ and 200,000 MJ ha^−1^ per year [10].

Regarding research results on the Jerusalem artichoke torrefaction process using a batch reactor, the findings of the torrefaction process temperature are quite important when designing commercial continuous reactors for the production of carbonized solid biofuels. These results indicate that, as is well known in the production of carbonized solid biofuels, in order to obtain the best process conditions of the torrefaction process and a reasonable price of the final product, it is important to achieve a mass loss on a level of 30% and an energy loss (torgas) on a level of 10% in the thermo-chemical conversion. Compared to research results on other energy crops and straw biomass, Jerusalem artichoke’s temperature of 245 °C during torrefaction for carbonized solid biofuel production under isothermal conditions is relatively low [41,42,43,44,45,46,47,48,49]. Research on the Jerusalem artichoke has shown that the amount of ash after the torrefaction process is still at a relatively low level compared to biomass not subjected to the torrefaction process (Jerusalem artichoke unprocessed as a result of the torrefaction process has an ash content of <3%), and solid fossil fuels, such as Polish hard coal, have an ash content of <15%. An SEM-EDS analysis of the ash composition of torrefied Jerusalem artichoke after burning at 700 °C showed a very favorable composition of mineral substances that can be reused as additives to organic fertilizers, a carrier of such elements as K (20.46%) and P (3.36%) and C (22.51%).

An SEM-EDS analysis was performed on fly ash of Jerusalem artichoke sintered at 700 °C.

The method of growing Jerusalem artichoke presented in the experiments carried out is ecological and improves the parameters of the torrefaction process. The Jerusalem artichoke produced as a result of ecological fertilization is characterized by low cultivation costs and the heat energy produced in this carbonized solid biofuel requires about 25% less expenditure than during fertilization with chemical fertilizers.

## 4. Materials and Methods

### 4.1. Plants, Waste, and Biopreparations

The bulbs of Jerusalem artichoke used in the experiments were purchased from Chmiel Ecological Farm (Poland). The non-centrifuged liquid waste was obtained from Gamawind Sp. z o.o., Piaszczyna, Poland, a distillery integrated with the biogas plant, which produces alcohol and biogas using corn grain as raw material. Apol-humus, the new generation soil improver, was purchased from the manufacturer Poli-Farm Sp. z o.o., Poland, whereas Stymjod, a nano-organic-mineral fertilizer, was supplied by the producer PHU Jeznach Sp. J., Poland.

### 4.2. Ultimate Analysis

The ash content of the raw samples of Jerusalem artichoke was measured using an electrical oven using the standard procedure. A main assumption was taken that no ash is lost during the torrefaction stage. Therefore, an ash content value was calculated for each solid residue from the overall mass yields. The C, H, N, and S contents were measured using a Perkin/Elmer Analyser and the elemental analyses procedure was used. All three different samples of Jerusalem artichoke were analyzed: Measurements were repeated in triplicate and the average value, which was corrected for moisture content, is presented in Table 1.

### 4.3. Plant Treatments and Experimental Design

The research was performed in north Poland in a field where the temperature oscillates from 11 to 21 °C, precipitation is 655 mm, and moist air from the Baltic Sea is noted. The experiments were carried out in podzolic soil, on 7 plots, which were fertilized in April with:

— Non-centrifuged waste from a corn grain biodigestion to methane process in dosages of 0, 10, 20, 30, and 40 m^3^ ha^−1^;
— Non-centrifuged waste, 40 m^3^ ha^−1^ together with Apol-humus (10 L ha^−1^); and
— Non-centrifuged waste, 40 m^3^ ha^−1^ together with Apol-humus (10 L ha^−1^) and Stymjod (5 L ha^−1^).

**Figure 12:**
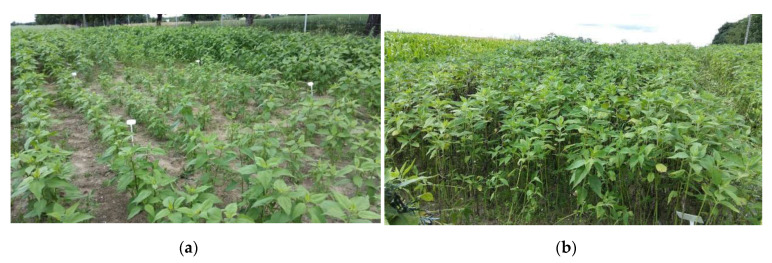
Jerusalem artichoke plants fertilized with various doses of biogas plant waste in Piaszczyna, (north Poland). The plot on the left fertilized with the dose of 10 m^3^ ha^−1^, in the middle the control (0 m^3^ ha^−1^ and on the right 40 m^3^ ha^−1^. Photo was taken on June 15 (**a**) and 2 of July (**b**).

All the experimental variants and elemental characteristic of wastes and biofertilisers are shown in Figure 1, Figure 2, Figure 3 and Figure 4 and Table 1 and Table 5. Waste and Apol-humus were mixed with the soil after their application while Stymjod was applied twice to leaves in July at a two-week interval. Jerusalem artichoke tubers were planted in the soil (enriched previously in April with waste and Apol-humus) in the first 10 days of May, 10–15 cm deep, 50 cm apart in a row, and 70 cm between rows, as is recommended. Non-fertilized plots/plants served as the control (Figure 12). The applied dosages of waste, Apol-humus, and Stymjod were chosen on the basis of previous research performed in a laboratory, container area, and field [19]. Jerusalem artichoke biomass was collected in November, evaluated for fresh and dry biomass, then chopped in a chopper and torrefied.

### 4.4. Fuel Characteristics

#### 4.4.1. Caloric Value

The caloric value was determined by using two methods. The first one was performed by using a calorimetric bomb and the second one by calculating the CV based on the carbon, hydrogen, and nitrogen content. One type of equation for CV calculation was used to find the value, called the partial least squares regression (PLS) method (Equation (1)), and this gave a higher heating value (HHV) on a dry basis. HHV was calculated using the formula:

(1) $$\mathrm{HHV}(\mathrm{PLS})=5.22C^{2}-319C-1647H+38.6\mathrm{CH}+133N+21028$$

where C = carbon, H = hydrogen, and N = nitrogen content expressed on a dry mass percentage basis. All caloric values from numerical calculations are the medium values of the determined results and are finally corrected by the dry ash content on a free basis.

The methodology of the experimental analysis of the Jerusalem artichoke torrefaction process for carbonized solid biofuel production comprised several analytical techniques:

— Analysis of the TGA, DTA, TG-FTiR, and TG-MS torrefaction process and biomass co-firing;
— Elemental analysis of biomass torrefaction process products;
— Analysis of the gases formed as a result of torrefaction: FTiR analysis and MS analysis;
— Technical analysis of biomass torrefaction products; and
— SEM-EDS ash analysis of torrefied Jerusalem artichoke after combustion.

**Figure 13:**
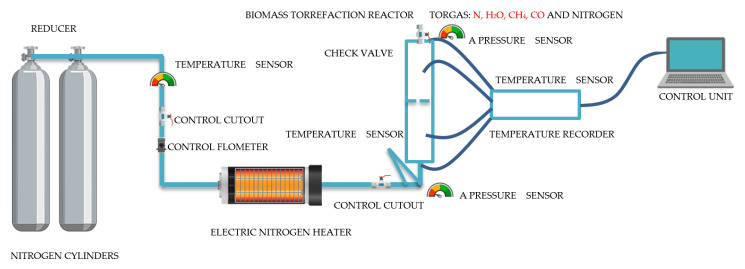
Scheme: installation with a batch reactor for the Jerusalem artichoke torrefaction process using nitrogen.

Experimental research on the torrefaction process of carbonized energy crops using a specially designed biomass torrefaction installation with a batch reactor was performed in an inert atmosphere, nitrogen (Figure 13). 

During biomass decomposition, three zones were distinguished on the weight loss curves of wood during torrefaction using installation with batch reactor Figure 12. The first one corresponded to the most reactive component, hemicellulose, whose decomposition started at 225 °C and finished at 325 °C; the second one was cellulose, whose decomposition temperature rate is from 300 °C up to 375 °C; and the last one was lignin, which represents the widgets’ temperature rate of 250–500 °C. The carbonization process of lignocellulosic biomass was described by weight loss kinetics by using different experimental devices. Among those many devices were fluidized bed reactors, thermogarvimetric analyzers, and tube furnaces. In the research presented, a method with a thermogravimetric analyser (TGA) was chosen to determine the weight loss kinetics of energy crop torrefaction. By using this kind of experimental method, we obtained dynamic conditions in which the sample with biomass was placed at a specific heating rate, but it is important to know that experimental heating rates are very often slower than those in real process equipment, such as combustors, reactors, or gasifiers.

#### 4.4.2. Ash Analysis of Torrefied Jerusalem Artichoke

The samples were investigated by scanning electron microscopy (SEM) and energy-dispersive X-ray spectroscopy (EDX) using an SEM FEI Quanta 200FEG microscope equipped with an EDX Oxford X-Max spectrometer. Measurements by the EDX technique were performed in at least 10 different spots for a given sample, and then an average atom concentration and its standard deviation were calculated for each of the identified elements. The electron energy of 20 keV was used in investigations.

### 4.5. Assessments of Plant Physiological Activity and Growth

The effects of fertilization treatments were assessed by periodical height measurements of whole plants during the vegetative season and evaluation of their biomass yield and energy value in the autumn. Assessments of the physiological activity of plants (gas exchange, index of chlorophyll content, enzyme activity) were carried out on fully developed leaves situated under the top of plants. In each experimental variant, one leaf from each of 10 plants was taken for the evaluation of gas exchange and enzyme activity. The material was collected in the third week of July in a temperature range of 25–30 °C, in sunshine and air humidity of 50–60%.

The height of the plants was measured at monthly intervals throughout the growing season [17]. The weights of fresh green biomass, and dry (dried at 130 °C for 3 days) were assessed in November on the basis of 5 plants taken from each experimental variant. The data presented were calculated for one plant as an average for the treatment [17]. Assessments of gas exchange (net photosynthesis, transpiration, stomatal conductance, and intercellular CO_2_ concentration) were performed using a TPS-2 -Portable Photosynthesis System (PP Systems, Amesbury, MA, USA) [17,20].

An index of the chlorophyll content in leaves was estimated using a SPAD-502 chlorophyll meter (Konica Minolta, Osaka, Japan) [17]. The activities of acid (pH 6) (EC 3.1.3.2) and alkaline (pH 7.5) (EC 3.1.3.1) phosphorylases (U g^−1^ (FM) min^−1^) in leaves and RNase (EC 3.1.27.5) (U g^−1^(FM) min^−1^) were studied using the methods demonstrated by Knypl and Kabzinska [50]. The activity of total dehydrogenases (EC 1.1.1.-) was measured using the procedure presented by Górnik and Grzesik [20,51] with a spectrophotometer (UVmini-1240, Shimadzu, Japan) for formazan determination at a wavelength of 480 nm.

### 4.6. Assessments of Biomass Energy Properties and Torrefied Materials

The energy crop samples were pre-prepared before the experiments, and the biomass was separated from foreign bodies, cleaned from contamination, chopped, and ground so as to reach the appropriate geometric dimensions. The plants were cut into sections of 2 to 4 cm, ground, and then sieved on a special automatic screen and dried in an electric oven at 110 °C for 4 h. Then, the samples were tightly closed and sent for technical and elemental analysis. The content of the elements carbon, hydrogen, nitrogen, and sulphur were determined and the volatility, moisture content, ash, combustion heat, and calorific values were determined (the same analysis was performed after the torrefaction process). The weight of the samples was determined before and after the drying process and the results were used to determine the moisture content. In each Jerusalem artichoke torrefaction process experiment, the dried biomass was divided into three separate samples of 20 g and each of them was evenly distributed on three horizontal screens of a reactor metal structure with different perforations made of acid-proof steel. The nitrogen was heated up by using electrical heaters to the set temperature (for this experimental research, temperatures between 220 °C and up to 280 °C were measured and the torrefied products were analyzed according to the mass loss and caloric value increase). Only selected samples were analyzed because, as a result of previous studies, a too high weight loss of more than 35% results in a very high degree of carbonization and high energy loss (above 10% of original energy content). The reverse is achieved with a mass loss below 25%, which is too low, and the biomass, in the end, is not fully roasted (it is not fragile and still has a high degree of moisture absorption and low calorific value).

### 4.7. Statistical Analysis

The investigations presented were performed in the field for three years (in a series) in northern Poland in three replicates for each experimental variant. The experimental plots and replicates, with differently fertilized plants, were situated randomly. Because of the similar climate conditions and growth in subsequent years, the data obtained were presented as means from the years and 10 plants (measuring height, gas exchange, index of chlorophyll content, enzyme activity) or 5 plants (taking weights of fresh green biomass) from each replicate. These were processed applying analysis of variance (ANOVA I), by Statistica 12. The means of the chosen parameters were grouped employing the Dunett’s test and the contrast between the control sample and the remaining samples was used at the α = 0.05 significance level.

## 5. Conclusions

The research presented shows the prospects of increasing the energy efficiency of Jerusalem artichoke crops by ecological use of the waste from corn grain biodigestion to methane, applied separately or together with Apol-humus, a new generation soil improver, and Stymjod, a nano-organic-mineral fertilizer, as an alternative to artificial fertilizers, which pollute the environment.

**Figure 14:**
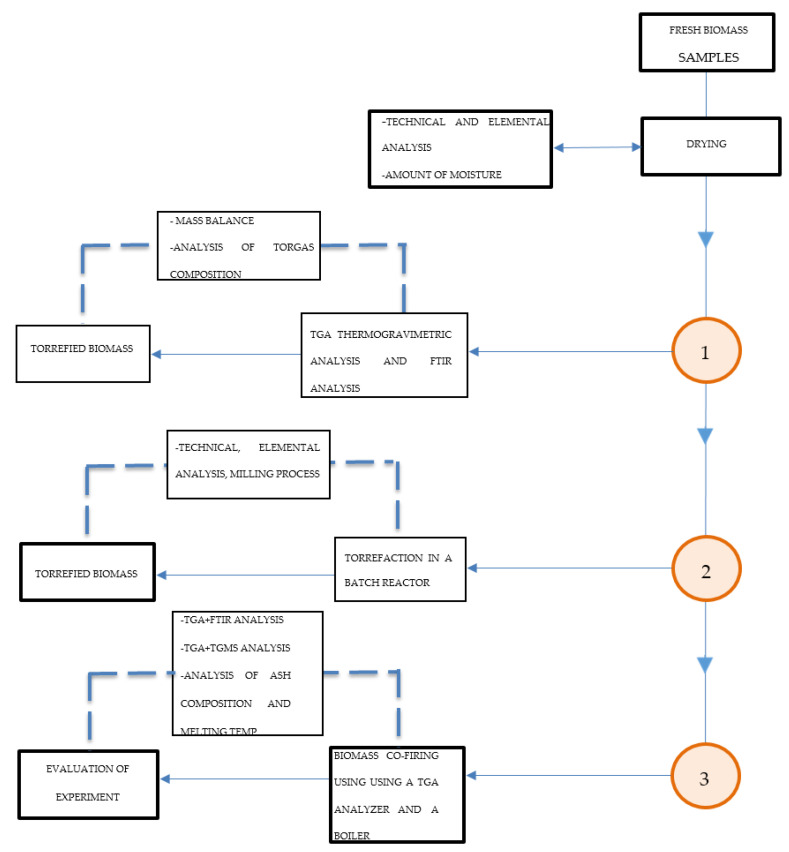
Scheme of the Jerusalem artichoke torrefaction process analytical method order: TGA, DTA, TG-FTiR, TG-MS torrefaction process, and biomass combustion plus SEM-EDS ash analysis.

The research presented shows that the temperature at which we obtained a 30% weight loss and a 10% energy loss as a result of the Jerusalem artichoke torrefaction process is 245 °C. The research shows an increase in the calorific value as a result of the Jerusalem artichoke torrefaction process from 15.82 to 22.12 MJ kg^−1^. Research on the Jerusalem artichoke torrefaction process Figure 14 has shown that the amount of ash after the torrefaction process in Jerusalem artichoke is still at a relatively low level compared to biomass not subjected to the torrefaction process (Jerusalem artichoke unprocessed as a result of the torrefaction process has an ash content of <3%) and solid fossil fuels, such as Polish hard coal, has an ash content of <15%. An SEM-EDS analysis of the ash composition after burning at 700 °C of torrefied Jerusalem artichoke showed a very favorable composition of mineral substances that can be reused as additives to organic fertilizers, a carrier of such elements as: K (20.46%) and P (3.36%) and C (22.51%).

An SEM-EDS analysis was performed on the fly ash of Jerusalem artichoke sintered at 700 °C and leaching toxicity of heavy metals was analyzed by a horizontal vibration extraction procedure (HVEP). It was found that the structure of fly ash is strengthened with an increase of the temperature, which is conducive to the stabilization of heavy metals.

The method of growing Jerusalem artichoke presented in the experiments carried out is ecological and improves the parameters of the torrefaction process. The Jerusalem artichoke produced as a result of ecological fertilization is characterized by low cultivation costs and the heat energy produced in this carbonized solid biofuel requires about 25% less expenditure than during fertilization with chemical fertilizers.

The research also demonstrates that biomass obtained under the influence of such fertilization can be used for energy purposes and, for example, can be converted to high energy density torrefied solid biofuel. It was demonstrated that Jerusalem artichoke could be a promising high-yielding energy crop. Its ecological fertilization with the waste from biogas plants (30–40 m^3^ ha^−1^), Apol-humus, and Stymjod positively influenced gas exchange (net photosynthesis, transpiration, stomatal conductance, and intercellular CO_2_ concentration), the index of chlorophyll content, the activity of the selected enzymes (acid and alkaline phosphorylase, RNase, and dehydrogenase), and energetic parameters, which markedly affected the Jerusalem artichoke growth kinetics during the whole vegetative season as well as the yield of fresh and dry biomass. The waste studied can be used as a cost-effective and environmentally friendly biofertilizer, if it is applied in defined doses and in agreement with the national legal regulations on the safe application of these components. The research shows that a torrefaction process temperature of around 245 °C is one of the most optimal temperatures for the production of carbonized solid biofuel from Jerusalem artichoke. Compared to research results on other energy crops and straw biomass, an isothermal temperature of 245 °C during torrefaction for carbonized solid biofuel of Jerusalem artichoke biomass fertilized with biogas plant waste is relativlely low. In the near future, biomass plants will be a major source of biofuel production and may inhibit the growth of oil prices [52].

## Figures and Tables

**Table 1:** Activities of the selected enzymes in leaves of Jerusalem artichoke plants grown in a field and fertilized with liquid non-centrifuged waste from corn grain digestion to methane (E10, 40 m^3^ ha^−1^), Apol-humus (AH; 10 L ha^−1^), and Stymjod (S; 5 L ha^−1^).

Waste Doses and Biopreparations Applied to Soil	Phosphorylases		RNase [U g^−1^ f.w.]	Total Dehydrogenases [mg Formazan g Leaf^−1^]
	(pH = 6.0) [U g^−1^ f.w.]	(pH = 7.5) [U g^−1^ f.w.]		
Control	0.60 a	0.26 a	2.6 a	0.50 a
AH	0.63 b	0.28 b	2.8 b	0.52 b
S	0.66 c	0.31 c	3.2 c	0.66 c
E10	0.63 b	0.28 b	2.9 b	0.52 b
E20	0.67 c	0.31 c	3.3 c	0.66 c
E30	0.71 de	0.33 de	3.6 de	0.69 d
E40	0.73 e	0.34 e	3.7 e	0.72 e
E40 + AH	0.77 f	0.39 f	4.2 f	0.76 f
E40 + AH + S	0.81 g	0.42 g	4.4 g	0.79 g
LSD_0.05_	0.02	0.01	0.1	0.15

The data marked with the same letters within a column are not significantly different, according to a Newman–Keuls multiple range test at an alpha level of 0.05. The data presented are the average over the years and 10 plants in each repetition of a particular experimental variant.

**Table 2:** Experimental research results of the Jerusalem artichoke torrefaction process in nitrogen using a batch reactor.

Sample Number	Mass Reduction,g	Mass Loss,%	Residential Time, min	Torrefaction Temp., °C
1	20/14, 64	26, 80	12	251, 17
2	20/14, 38	28, 10	13	245, 17
3	20/13, 47	32, 65	14	247, 90
4	20/14, 50	27, 50	13	242, 17
5	20/14, 21	28, 95	14	243, 45
6	20/12, 51	37, 45	17	254, 06
7	20/15, 26	23, 70	10	241, 64

**Table 3:** Elemental analysis and technical analysis of Jerusalem artichoke before and after the torrefaction process.

Energy Crop	Moisture(%)	C ^ad^, (%)	N ^ad^, (%)	H ^ad^, (%)	S ^ad^, (%)	Cl, (%)	Volatile ^ad^ (%)	Ash (%)	High Heating Value, (^MJ^/_kg_)
Jerusalem artichoke	5.3	48.5	0.27	6.20	0.05	0.115	91.29	2.3	15.82
Torrefied Jerusalem artichoke:									
(243, 45 °C, 14 min)	2.8	54.37	0.19	5.37	0.05	0.014	73.37	3.94	21.70
(245, 17 °C, 13 min)	2.7	55.04	0.19	5.34	0.05	0.014	72.81	3.84	22.12
(242, 17 °C, 13 min)	2.8	54.79	0.19	5.39	0.05	0.014	72.27	3.71	22.09

^ad^ Add dry basis.

**Table 4:** Content of elements in the ash from untreated Jerusalem artichoke and torrefied Jerusalem artichoke burnt biomass.

Assessed Material	C	O	K	Ca	Mg	Fe	Si	P	S	Cl	Dry Mass
		[Atomic, %]									[%]
Ash composition from torrefied Jerusalem artichoke (average values)	21.75	46.82	23.01	2.63	0.81	0.02	0.34	3.02	0.19	1.41	100.00
Standard deviation, σ	3.00	2.18	2.83	0.51	0.30	0.00	0.10	0.60	0.05	0.40	- *
Ash composition from untreated Jerusalem artichoke (average values)	31.53	36.05	20.09	2.22	0.98	0.11	0.46	1.16	0.19	3.13	100.00
Standard deviation, σ											
	3.32	4.00	3.88	0.72	0.27	0.01	0.42	0.62	0.05	1.19	- *

* The values of standard deviations do not add up.

**Table 5:** Content of elements in the non-centrifuged waste from corn grain biodigestion to methane, Stymjod, and Apol-humus.

Assessed Material	pH	N	P	K	Ca	Mg	Fe	Mn	Cu	Zn	B	Dry Mass
		[mg L^−1^]										[%]
Waste	7.6	2455	278	996	300	115	9.0	0.324	0.175	0.976	3.365	1.4
Stymjod	5.4	1230	6650	62722	945	11574	18.7	885	680	1470	573	-
Apol-humus	12	15.21	15.8	20.2	468	70	140	5.95	0.87	2.40	0.92	-
